# HIF2α Upregulates the Migration Factor ODZ1 under Hypoxia in Glioblastoma Stem Cells

**DOI:** 10.3390/ijms23020741

**Published:** 2022-01-11

**Authors:** María Carcelén, Carlos Velásquez, Veronica Vidal, Olga Gutierrez, Jose L. Fernandez-Luna

**Affiliations:** 1 Genetics Unit, Hospital Universitario Marqués de Valdecilla, 39008 Santander, Spain; maria.carcelen93@gmail.com (M.C.); veronicavisan@gmail.com (V.V.); molga.gutierrez@scsalud.es (O.G.); 2 Instituto de Investigación Marqués de Valdecilla (IDIVAL), 39008 Santander, Spain; carvelhn@gmail.com; 3 Department of Neurological Surgery, Hospital Universitario Marqués de Valdecilla, 39008 Santander, Spain; 4 Department of Anatomy and Cell Biology, Universidad de Cantabria, 39008 Santander, Spain

**Keywords:** glioblastoma, hypoxia, glioblastoma stem cells, migration, ODZ1, HIF2α

## Abstract

**Background:** Glioblastoma (GBM) remains a major clinical challenge due to its invasive capacity, resistance to treatment, and recurrence. We have previously shown that ODZ1 contributes to glioblastoma invasion and that ODZ1 mRNA levels can be upregulated by epigenetic mechanisms in response to hypoxia. Herein, we have further studied the transcriptional regulation of ODZ1 in GBM stem cells (GSCs) under hypoxic conditions and analyzed whether HIF2α has any role in this regulation. **Methods:** We performed the experiments in three primary GSC cell lines established from tumor specimens. GSCs were cultured under hypoxia, treated with HIF regulators (DMOG, chetomin), or transfected with specific siRNAs, and the expression levels of ODZ1 and HIF2α were analyzed. In addition, the response of the ODZ1 promoter cloned into a luciferase reporter plasmid to the activation of HIF was also studied. **Results:** The upregulation of both mRNA and protein levels of HIF2α under hypoxia conditions correlated with the expression of ODZ1 mRNA. Moreover, the knockdown of HIF2α by siRNAs downregulated the expression of ODZ1. We found, in the ODZ1 promoter, a HIF consensus binding site (GCGTG) 1358 bp from the transcription start site (TSS) and a HIF-like site (CCGTG) 826 bp from the TSS. Luciferase assays revealed that the stabilization of HIF by DMOG resulted in the increased activity of the ODZ1 promoter. **Conclusions:** Our data indicate that the HIF2α-mediated upregulation of ODZ1 helps strengthen the transcriptional control of this migration factor under hypoxia in glioblastoma stem cells. The discovery of this novel transcriptional pathway identifies new targets to develop strategies that may avoid GBM tumor invasion and recurrence.

## 1. Introduction

Glioblastoma (GBM), a grade IV glioma according to the WHO 2021 classification [1], is responsible for approximately 50% of all malignant primary tumors of the brain [2]. Despite multidisciplinary therapeutic approaches such as surgery, chemo- and radiotherapy, and combined therapies, the patients have a median survival of 14–15 months from diagnosis [3]. Its dismal prognosis makes it an essential public health problem and highlights the need to identify new therapeutic targets. 

It is widely accepted that, in addition to the high intra- and intertumoral heterogeneity [4,5,6,7] and the relevance of tumor microenvironment [6], glioblastoma stem cells (GSCs) have a fundamental role in the pathogenesis and recurrence of GBM [4,6]. GSCs have similar properties to stem cells, such as high plasticity, proliferation, and self-renewal [4,7,8], and, despite being only a slight proportion of total tumor cells, they have been recognized as key players in tumor angiogenesis and in the response to hypoxic microenvironments [9,10,11]. To this end, hypoxia enhances the migration of GSCs towards the peritumoral parenchyma and may contribute to the acquisition of chemoresistant mechanisms. Moreover, both hypoxia and GSCs contribute to generating an immunosuppressive state in the tumor microenvironment [10].

The infiltration of the surrounding parenchyma by tumor cells is responsible for recurrence in gliomas, and it is present in almost 100% of cases of GBM [5,11]. The processes of migration and invasion are dynamic and include adhesion to components of the extracellular matrix, the reorganization of the cytoskeleton, and the degradation of matrix proteins by tumor-secreted enzymes [8]. These processes are regulated by several pathways including PI3K/Akt, NFkB, Wnt/β-catenin, Rho GTPases, and some transcription factors, including STAT3 and C/EBPβ [12]. Nevertheless, despite that multiple inhibitors of these pathways have been developed [9], clinical trials have not yet yielded reliable and clinically significant results.

Previous studies from our group showed that ODZ1 (also known as teneurin-1, TENM1), which plays a key role in central nervous system ontogenesis [13], contributes to promoting the migration and invasion of GBM cells by inducing actin cytoskeletal remodeling through RhoA/ROCK activation [12]. We also showed that ODZ1 is upregulated in GBM cells through a Stat3-mediated pathway activated by IL-6, which is released by tumor-associated monocytes [10]. In addition, a hypoxic microenvironment regulates GBM tumor cell migration in part by inducing ODZ1 through hypomethylation of a CpG island in the ODZ1 promoter [14].

Taking into consideration that the main and best known elements that mediate the response of cells to hypoxia are the hypoxia-inducible factors (HIF) [15], we wanted to investigate if they have any role in the transcriptional regulation of ODZ1. Here, we present novel results showing the increased expression of ODZ1 by the transcription factor HIF2α in GSCs cultured under hypoxia.

## 2. Results

### 2.1. Upregulation of ODZ1 mRNA in Response to Hypoxia Correlates with Increased Protein Levels of HIF2α

**Figure 1:**
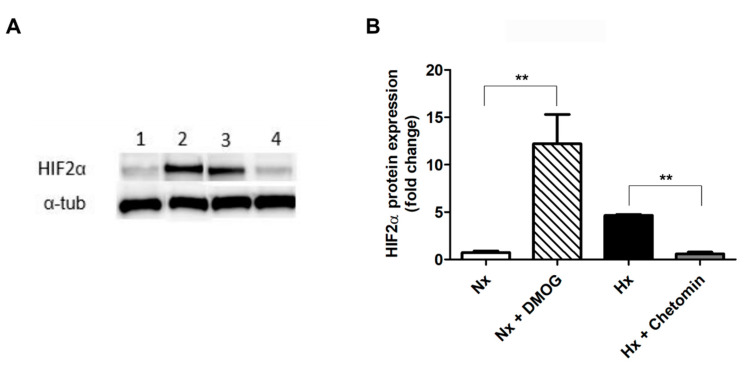
Expression of HIF2α protein is increased under hypoxic conditions. (**A**) GSCs were cultured under different conditions, and the HIF2α expression was analyzed by Western blot in total cell lysates (a representative image is shown). 1: normoxia; 2: normoxia + DMOG; 3: hypoxia; 4: hypoxia + chetomin. (**B**) Image band quantification by using ImageJ analysis software. Nx: normoxia; Hx: hypoxia. Data were normalized to α-tubulin levels. ** *p* < 0.01. Bars represent mean ± SEM of three independent experiments.

It has been described that HIF2α can be induced in GSCs and is associated with the maintenance of stem-like properties [16]. The gene silencing of HIF2α compromises GSC phenotype, induces apoptosis, and inhibits cell growth and angiogenesis [16,17]. Based on this, we studied the role of HIF2α in the expression of ODZ1 in three GSC cell lines established from the tumor specimens of patients with GBM (G63, G178, and G196). We cultured GSCs under hypoxia (1% oxygen) or in the presence of HIF regulators DMOG (a stabilizer of the HIFα protein) and chetomin (a blocker of the HIF pathway). HIF2α protein levels were upregulated under hypoxia and decreased when cells were treated with chetomin (Figure 1). In addition, HIF2α protein increased in the presence of DMOG in normoxia.

**Figure 2:**
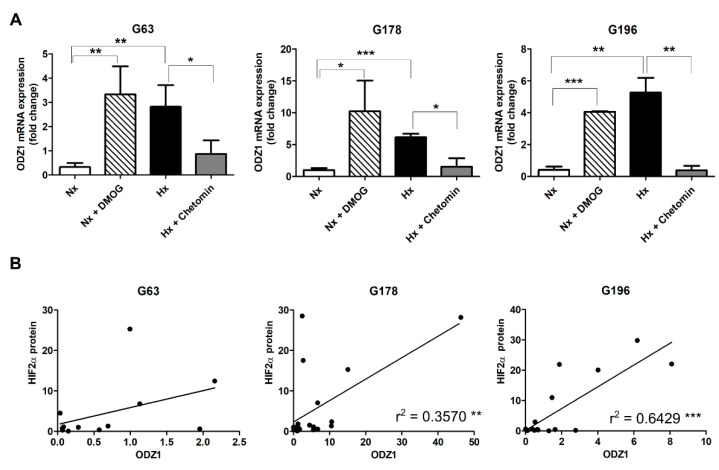
GSCs expressed ODZ1 in response to hypoxia and HIF regulators. (**A**) GSC cell lines were cultured in normoxia (Nx, 21% O_2_) or hypoxia (Hx, 1% O_2_) and treated or not with 250 nM chetomin or 0.5 µM DMOG. ODZ1 expression was analyzed by qPCR. Data were normalized to G6PD levels. * *p* < 0.05, ** *p* < 0.01, *** *p* < 0.001. Bars represent mean ± SEM of three independent experiments. (**B**) Correlation between ODZ1 mRNA and HIF2α protein. Pearson’s coefficient (r); *p* < 0.001.

The same experimental conditions were used to analyze the mRNA expression of ODZ1 in all three GSC cell lines (Figure 2A). In all cases, DMOG increased more than 10-fold the levels of ODZ1 in normoxic cells. Similar ODZ1 upregulation was obtained in GSCs cultured under hypoxia, which was drastically reduced in the presence of chetomin (Figure 2A). Interestingly, there was a good positive correlation between the protein levels of HIF2α and the expression of ODZ1 mRNA (Figure 2B).

### 2.2. Positive Correlation between the Relative mRNA Expression of ODZ1 and HIF2α in GSCs

**Figure 3:**
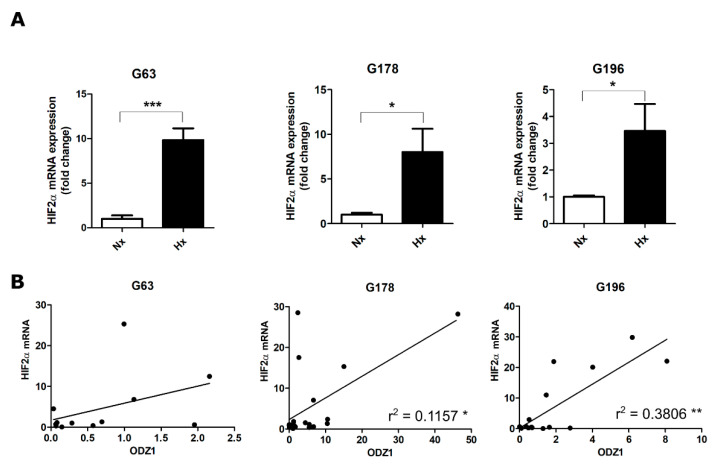
Expression of HIF2a mRNA correlates with ODZ1 mRNA levels. (**A**) GSC cell lines were cultured under hypoxia, and the expression of HIF2α mRNA was analyzed by qPCR. Data were normalized to G6PD levels. * *p* < 0.05, ** *p* < 0.01, *** *p* < 0.001. Bars represent mean ± SEM of three independent experiments. (**B**) Correlation between ODZ1 mRNA and HIF2α mRNA. Pearson’s coefficient (r); *p* < 0.001.

Classically, it has been considered that hypoxia increases the protein levels of HIFα, and only a few studies have observed the regulation of HIFα at the mRNA level. Similar to our previous experiments, GSC cell lines were cultured under hypoxia conditions. In all cell lines, hypoxia increased HIF2α mRNA more than 3-fold when compared with normoxic cells (Figure 3A). Consistent with our previous results with HIF2α protein, there was a positive correlation between the mRNA levels of ODZ1 and those of HIF2α (Figure 3B).

### 2.3. Knocking Down HIF2α Reduces the Expression of ODZ1 in GSCs

**Figure 4:**
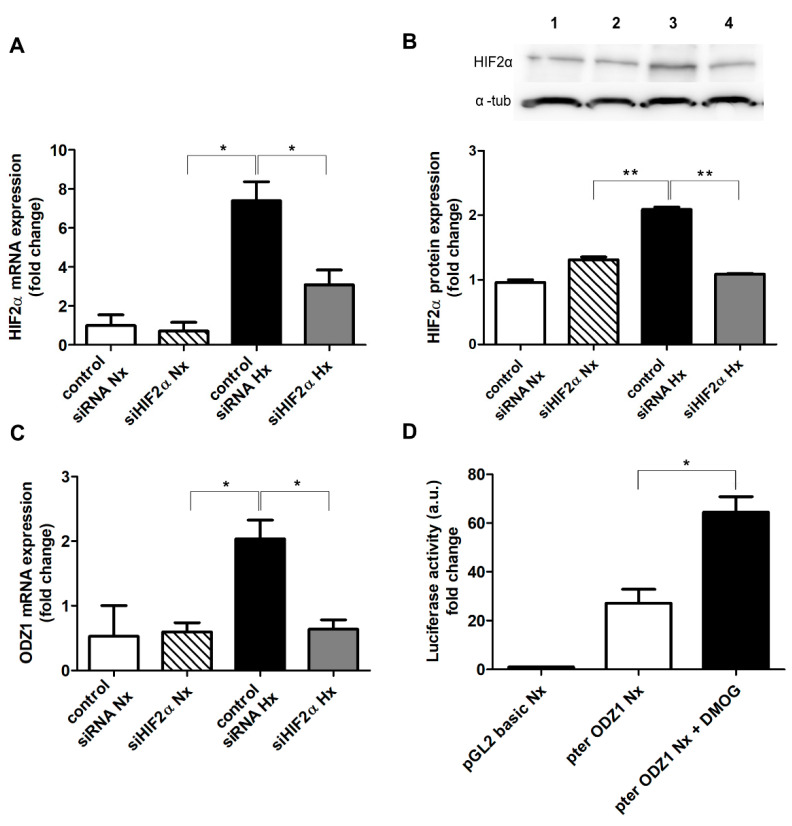
Knockdown of HIF2α decreased the expression of ODZ1. (**A**,**B**) GSCs were transfected with siHIF2α or siRNA control and cultured in normoxia (Nx, 21% O_2_) or hypoxia (Hx, 1% O_2_) for 48 h. The expression of HIF2α at the mRNA (**A**) and protein (**B**) level was analyzed. 1: negative control siRNA in normoxia; 2: siHIF2α in normoxia; 3: negative control siRNA in hypoxia; 4: siHIF2α in hypoxia. Data were normalized to G6PD levels (mRNA) and α-tubulin (protein). * *p* < 0.05, ** *p* < 0.01. (**C**) Under the same experimental conditions, the mRNA levels of ODZ1 were also quantitated by qPCR. (**D**) GSCs were transfected with ODZ1 promoter cloned into a luciferase reporter plasmid and luciferase activity was analyzed after 48 h in the presence or in the absence of DMOG under normoxia. pGL2: empty plasmid (control). * *p* < 0.05. Bars represent the mean ± SEM of three independent experiments.

Given the positive correlation between HIF2α and ODZ1 in GSCs, we performed a gene silencing experiment. The transfection of GSCs with siRNAs specific for HIF2α reduced at least 2-fold both the mRNA (Figure 4A) and protein (Figure 4B) levels of HIF2α under hypoxia. Interestingly, the downregulation of HIF2α decreased the mRNA expression of ODZ1 (more than 2-fold) in hypoxic GSCs (Figure 4C).

To further confirm the transcriptional association between HIF2α and ODZ1 expression, we analyzed the ODZ1 promoter sequence and found a HIF consensus binding site (GCGTG) 1358 bp from the transcription start site (TSS) and a HIF-like site (CCGTG) 826 bp from the TSS. A promoter fragment of 1.4 kb cloned in a luciferase reporter plasmid was transfected into GSCs, and cells were cultured in the presence or in the absence of HIFα stabilizer DMOG. As shown in Figure 4D, DMOG increased more than 2-fold the promoter activity of ODZ1 in normoxic GSCs as determined by luciferase assays.

## 3. Discussion

It has been extensively described that hypoxia stimulates the growth and migration of GSCs. This point may be critical for recurrence since GSCs that remain in the infiltrated parenchyma after surgery are able to interact with their microenvironment to form a new tumor. Therefore, the identification of molecular pathways involved in GSC migration and invasion remains crucial in order to find new therapeutic targets. Based on in vitro and in vivo experiments, we previously showed that ODZ1 greatly contributes to the migration and invasion of glioblastoma cells, including GSCs [12]. We also described how ODZ1 mediates, at least in part, the hypoxia-dependent tumor migration of these cells [14]. We hypothesized that the expression of a gene involved in promoting migration might be tightly controlled by cancer cells taking advantage of different transcriptional mechanisms.

Herein, we showed that ODZ1 is transcriptionally regulated by HIF2α, a member of a family of transcription factors widely known to mediate the response to hypoxia by inducing the expression of a number of target genes [15]. GSCs cultured under hypoxia conditions or treated with a stabilizer of the HIFα protein upregulated the expression of ODZ1. Of note, the expression of ODZ1 mRNA positively correlated with both the mRNA and protein levels of HIF2α. The classical view of HIF signaling describes the post-translational stabilization of the *alpha* subunit and its translocation to the nucleus and binding to the *beta* subunit. However, consistent with our result, some authors have described an increased transcription of *alpha* subunits under hypoxia [17,18].

The functional relationship between ODZ1 and HIF2α was further studied by knocking down HIF2α. The downregulation of this transcription factor led to decreased mRNA levels of ODZ1. Taking into account previous work showing that HIF2α promotes the expression of stem cell markers, including nestin and CD133 [16], it is likely that this transcription factor might contribute to maintaining the stem cell phenotype of GSCs and providing a mechanism to allow GSCs to migrate out of hypoxic microenvironments. We have identified two potential HIF binding sites within the promoter of ODZ1. Luciferase assays confirmed that ODZ1 promoter was activated in the presence of an HIFα protein stabilizer. In addition to this HIF2α-mediated transcriptional regulation of ODZ1, we previously described another mechanism that upregulates ODZ1 in GSCs in response to hypoxia. Hypoxic microenvironments induced the hypomethylation of a CpG island in the ODZ1 promoter, resulting in higher levels of ODZ1. Moreover, the mutation of this CpG island decreased the effect of hypoxia on ODZ1 expression [14]. Interestingly, some authors have previously described the epigenetic regulation of chromatin as another important regulatory mechanism of response to hypoxia [19].

The role of HIF1α in regulating several signaling pathways that result in tumor cell migration has been extensively described in previous reports, and it is widely accepted [20]. Nevertheless, the role of HIF2α in GBM cell migration has been scarcely reported [21]. It is widely accepted that HIF1α and HIF2α play partially overlapping roles in different types of cancer cells [22]. However, several reports point out the predominant role of HIF2α in pathogenic mechanisms in GCSs [17,23].

In conclusion, our results identify HIF2α as a transcriptional regulator of the migration factor ODZ1 in response to hypoxia, unraveling a novel signaling pathway as a potential target to avoid GSC migration towards non-hypoxic microenvironments, which may lead to tumor recurrence. This is in line with the main goals in GBM treatment that include approaches to overcoming the hypoxia-mediated radio- and chemoresistance and recurrence of GBM tumors and consequently, the dismal survival of GBM patients. Potential strategies include decreasing HIF protein expression and stabilization and preventing the binding of HIF to its consensus sites in the genome, thus inhibiting the expression of target genes. Whether strategies to block or reduce the expression of ODZ1 have a significant contribution to avoid recurrence of GBM needs further in vitro and in vivo studies. This and other similar approaches will pave the way towards more personalized management of this aggressive tumor.

## 4. Materials and Methods

Primary cell cultures: We used three primary GSC cultures, G63, G178, and G196, previously established in our laboratory [12,24]. Cells were maintained as stem-like neurospheres in a serum-free DMEM/F12 medium (Invitrogen, Carlsbad, CA, USA) and used within 10–20 passages. Cells were incubated under hypoxia (1% O_2_) in a Hypoxia Incubator Chamber (StemCell Technologies, Vancouver, Canada). When indicated, GSCs were treated with 0.5 mM DMOG or 250 nM chetomin (both from Sigma-Aldrich, St. Louis, MO, USA).

qPCR analysis: Quantitative RT-PCR was performed, in a 7000-sequence detection system (Applied Biosystems, Foster City, CA, USA), from total cellular RNA as previously described [12] We used the following primers: G6PD (5′-ATCGACCACTACCTGGGCAA-3′; 5′-TTCTGCATCACGTCCCGGA-3′), ODZ1 (5′-ACTCAAGAGATGGAATTCTGTG-3′; 5′-CTTAGTGCATGGTCAGGTG-3′) and HIF2α (5′-ATGACAGCTGACAAGGAGAAGA-3′; 5′-TGGGCCAGCTCATAGAACAC-3′).

Western blot analysis: Total protein lysates were obtained from GBM cells as described [18]. Blots were incubated with antibodies against HIF2α (BL-95-1A2, Bethyl laboratories, Montgomery, AB, USA) and α-tubulin (sc-23948, Santa Cruz Biotechnology, Dallas, TX, USA). Anti-rabbit and anti-mouse IgG antibodies conjugated to horseradish peroxidase (sc-2357 and sc-51610, Santa Cruz Biotechnology) were used as secondary antibodies.

Luciferase and gene silencing assays: Cells were transfected with ODZ1 promoter cloned into pGL2-luciferase reporter plasmid and pRSV-β-gal by using nucleofection [14]. After 48 h of transfection, the relative luciferase activity was evaluated by a dual-light reporter gene assay (Applied Biosystems). Results were normalized with the values obtained with pRSV-β-gal.

Cells were transfected with HIF2α siRNA or negative control siRNA (mission siRNA, Sigma-Aldrich) by using Lipofectamine RNAiMAX Transfection Reagent (Invitrogen, Waltham, MA, USA).

Stadistical Analysis: Data are presented as mean ± SEM of three independent experiments. Differences between groups were tested by unpaired two-tailed Student’s *t*-test. The significance level was set at *p* < 0.05. All statistical analyses were performed using GraphPad Prism 5.0 (GraphPad Software, Inc., San Diego, CA, USA).

## Data Availability

Not applicable.
